# Breaking the Habit? Identifying Discrete Dimensions of Sitting Automaticity and Their Responsiveness to a Sitting-Reduction Intervention

**DOI:** 10.1007/s12529-023-10155-4

**Published:** 2023-02-07

**Authors:** Benjamin Gardner, Casey P. Mainsbridge, Amanda L. Rebar, P. Dean Cooley, Cynthia Honan, Jane O’Brien, Scott J. Pedersen

**Affiliations:** 1 Department of Psychology, University of Surrey, Guildford, GU2 7XH Surrey UK; 2 School of Education, University of New England, Armidale, NSW 2351 Australia; 3 School of Health, Medical, and Applied Sciences, Central Queensland University, Rockhampton, QLD 4702 Australia; 4 Active Work Laboratory, School of Education, University of Tasmania, Newnham, TAS 7250 Australia; 5 School of Psychological Sciences, University of Tasmania, Newnham, TAS 7250 Australia; 6 School of Nursing, Queensland University of Technology, Kelvin Grove, QLD 4059 Australia

**Keywords:** Sitting, Sedentary behaviour, Habit, Automaticity, Behaviour, Health psychology

## Abstract

**Background:**

Growing evidence suggests that sitting is activated automatically on exposure to associated environments, yet no study has yet sought to identify in what ways sitting may be automatic.

**Method:**

This study used data from a 12-month sitting-reduction intervention trial to explore discrete dimensions of sitting automaticity, and how these dimensions may be affected by an intervention. One hundred ninety-four office workers reported sitting automaticity at baseline, and 3 months, 6 months, 9 months and 12 months after receiving one of two sitting-reduction intervention variants.

**Results:**

Principal component analysis extracted two automaticity components, corresponding to a lack of awareness and a lack of control. Scores on both automaticity scales decreased over time post-intervention, indicating that sitting became more mindful, though lack of awareness scores were consistently higher than lack of control scores.

**Conclusion:**

Attempts to break office workers’ sitting habits should seek to enhance conscious awareness of alternatives to sitting and afford office workers a greater sense of control over whether they sit or stand.

## Introduction

Sitting time has been linked with increased mortality and morbidity [1, 2], and engaging in the recommended weekly 150 min of physical activity may not offset health detriments [3, 4]. Prolonged sitting poses cardiometabolic risks, and breaking up sitting bouts can reduce such risks [5–7]. Desk-based office workers are of especial concern [8]: likely due to the sitting-conducive nature of typical office environments and work tools [9], they typically sit for around two-thirds of their working day [10, 11]. Over a third of office workers report sitting for more than 30 min per bout [12]. Interventions are needed to break up sitting time among office workers [13].

The development of effective interventions will be aided by identifying modifiable psychological antecedents of prolonged sitting. While some researchers have sought to portray sitting as the product of conscious reasoning [14], evidence increasingly suggests that sitting is undertaken with minimal forethought or deliberation [15–17]. A more nuanced appraisal is that sitting arises through both conscious and non-conscious processes [18]. Sitting may perhaps best be understood within a psychological structure of hierarchically organized action and outcome goals [19–22]. The abstract, personally meaningful outcome goals situated at higher levels of the hierarchy (e.g. ‘get a promotion at work’) direct the activation of lower-level actions that serve those outcome goals (e.g. ‘complete my work tasks’). At the lowest level of the hierarchy are mechanistic actions that enable performance of higher-order actions (e.g. ‘use my work computer’). Studies of how people conceive of sitting suggest that the act of sitting occupies a relatively low level in this hierarchy [21]. Sitting per se lacks inherent meaning to office workers and is incidentally incurred in pursuit of more meaningful goals [21]. Importantly, actions at lower levels of the goal hierarchy are less likely to demand or receive conscious attention. Sitting at work is likely prompted automatically, on exposure to associated office-based cues, such as arriving in the office; that is, for many office workers, sitting is habitual [16, 23].

Habitual behaviours are learned through context-dependent repetition: repeatedly enacting a behaviour (e.g. sitting) following exposure to a cue (e.g. entering the office) leads, through associative learning, to the action being activated automatically upon subsequent exposure to the cue [24, 25]. As behaviour becomes habitual, control over initiation of the action is transferred to external cues and automaticity, and dependence on conscious motivational processes lessens [26]. Automaticity is the ‘active ingredient’ of habit [27]: by virtue of its automatic activation, habit can sustain familiar actions like sitting even when people do not consciously intend to do them, and potentially despite intending *not* to do them [28, 29]. Automaticity has multiple facets [30]: an action can be said to be automatic when it proceeds in a cognitively efficient way, or when the actor lacks awareness of the action, intention to perform the action or control over the action [31]. It is, however, not necessary for all four facets to be present for an action to be automatic. The goal-directed nature of sitting, for example, is such that, to the extent that it is undertaken in the pursuit of higher-order goals, it is likely to be aligned with intentions [30, 32], yet may also be done efficiently and outside of awareness.

Identifying in what respect sitting is automatic may help develop understanding of the specific mechanisms through which interventions may operate, and so aid design of effective interventions to break sitting habits. The dominant measure of habit-related automaticity over the past 20 years has been the Self-Report Habit Index (SRHI) [33]. The index comprises 12 items with which participants rate their agreement, which are designed to capture the automaticity (“[Behavior X—e.g., ‘sitting’] is something…”; ‘…I do automatically’), repetition history (‘…I have been doing for a long time’) and self-identity relevance of action (‘…that’s typically me’). Repetition history, assessed by three SRHI items, is a precursor and consequence of habit-related automaticity but not a facet of automaticity itself, and self-identity, assessed by one item, may correlate with but is not a core component of automaticity [34]. Removing these leaves eight items intended to capture automaticity. Attempts have been made to reduce these items further, most notably within the Self-Report Behavioural Automaticity Index, which comprises a set of four SRHI items proposed to assess behavioural automaticity most parsimoniously [35]. However, the authors of the SRHI [36] argue that the full eight items are needed to comprehensively capture the four automaticity facets proposed by Bargh [31]: lack of cognitive effort (‘…I do without having to consciously remember’), lack of awareness and conscious intent (‘…I do without thinking’) and lack of control (‘…I start doing before I realize I’m doing it’) [33]. These automaticity items are typically shown to have a unidimensional structure, rather than being discernible into three separable dimensions of automaticity [37, 38]. This may reflect that some of the items are conceptually blurred; for example, the item ‘sitting is something that would require effort not to do’ appears to incorporate aspects of both cognitive effortlessness and lack of control. Nonetheless, some studies have identified multiple dimensions within the SRHI. A study of responses to the 12-item SRHI in relation to physical activity and snacking behaviour found not only a separable behavioural frequency factor, but parsed automaticity into two factors, respectively corresponding to lack of awareness and lack of control [39].

Previous research has established that sitting is often automatic, but to our knowledge, no research has yet sought to explore in what ways it may be automatic ([18, 40, 41]; but see [42]). Using data from a 12-month trial comparing two variants of an intervention designed to break office workers’ sitting habits [43, 44], the present study examined the factor structure of the SRHI and changes in these factors following intervention. Both intervention variants centred on computerized, in-the-moment prompts designed to raise awareness of prolonged sitting and encourage movement breaks [45]. The present study represents secondary analyses of the trial data; primary analyses of intervention effectiveness have been reported elsewhere [46]. The aims of the present study were to, firstly, examine the internal structure of SRHI automaticity items as they relate to sitting and, secondly, to document how scores on discrete components extracted from this analysis changed over time in response to an intervention designed to disrupt prolonged sitting time. We expected sitting automaticity strength to decline in response to the intervention, but given the exploratory nature of the study, we did not formally hypothesize how many components would emerge, nor whether there would be differences in the responsiveness of any such components to the intervention.

## Method

### Participants and Procedure

Data were collected as part of a 12-month randomized trial comparing two workplace sitting-reduction interventions. Participants were desk-based employees from a state government department in Australia. Study inclusion criteria were as follows: (1) full-time employee with primarily desk-based job responsibilities, (2) used a personal computer with Internet access to perform work, (3) deemed medically healthy by completion of a Physical Activity Readiness Questionnaire (PAR-Q) [47] and (4) available for a 12-month period, to complete data collection requirements at baseline, 3 months, 6 months, 9 months and 12 months post-baseline.

Participants were recruited via a company-wide email, sent to 3922 recipients, offering them the opportunity to trial an e-health intervention designed to interrupt prolonged bouts of occupational sitting. A total of 595 employees across the state expressed interest, of whom 370 indicated that they would be willing to attend an initial orientation session that was a prerequisite for study entry. Of these, 232 employees attended the orientation session.

Orientation sessions involved discussions of sitting and health, and sitting reduction strategies, including a preview of the intervention. At the end of the session, attendees were invited to take part in our 12-month study. The 194 participants who consented during these sessions were sent a follow-up email with a link to an online survey assessing demographics and sitting automaticity. After completing the survey, participants were randomly allocated with replacement to receive one of two intervention variants, involving computer-automated prompts to reduce sitting. Intervention software was installed on each participant’s work computer the night before commencement of the intervention period. Participants in both groups were sent follow-up surveys by email every 3 months until 12 months post-baseline.

A total of 106 participants were allocated to the ‘active prompt’ group (93 female [88%], 13 male [12%]; mean age 44.35 years [SD = 10.83 years, range 23–71 years]) and 88 to the ‘passive prompt’ group (80 female [86%], 13 male [14%]; mean age = 45.83 years [SD = 10.03 years, range 24–64 years]). Groups were matched such that they did not differ in age (*t*[197] = 1.00, *p* = .32) or gender (*χ*^2^ = .72, *p* = .83). All procedures were approved by the University of Tasmania Social Sciences Research Ethics Committee (ref #H0010875).

### Intervention

To cover employees’ disperse locations, the same orientation session was delivered four times, each in a different location, and each delivered by the same two members of the research team. The session took 1 h and consisted of a 10-min informative discussion of the adverse health effects of prolonged sitting; 40-min discussion, demonstration and review of strategies to interrupt sitting; and 10-min preview and description of the e-health intervention software (‘Exertime’) that participants would receive, to demonstrate how the software functioned on work computers. A brief question and answer period was offered at the end of each session.

For all participants, the Exertime software automatically initiated a prompt sequence every 45 min, via a small bubble that appeared in the bottom right-hand corner of the participant’s computer screen. By clicking a drop-down menu, participants allocated to receive the *passive prompt* intervention variant could choose to ‘Exertime now’, so triggering an on-screen sequence promoting taking a movement break, or postpone their Exertime for 5 min, 10 min or 15 min. Those allocated to the *active prompt* variant had the same options, but crucially, had the additional option to ignore the Exertime prompt. The Exertime sequence involved a new, immovable screen being generated, which covered the participant’s current display, and which instructed them to interrupt their sitting and stand or move for a participant-selected time duration. In both conditions, computer-recorded daily cumulative data on the number of prompts accepted, i.e. all prompts in the passive prompt condition, and all non-ignored prompts in the active prompt condition, was visually fed back to participants, so that personal daily progress could be self-monitored throughout the workday.

### Measures

*Sitting automaticity* was self-reported, at five timepoints (baseline [time 1, T1] and 3 months [T2], 6 months [T3], 9 months [T4] and 12 months [T5] post-baseline), using six items from the SRHI [33], adapted to a sitting context. All 12 SRHI items were originally recorded for the broader intervention evaluation project. Authors BG and AR, who have especial expertise in habit theory and application, were invited to collaborate on the present study after the data were collected, and recommended post hoc reduction of the SRHI items due to concerns about their conceptual and construct validity. Specifically, two items relating to behavioural frequency (e.g. ‘sitting is something I do frequently’) were excluded, and so too was an item capturing identity relevance (‘sitting is something that’s typically me’), as these do not capture automaticity [34, 35]. Three potential automaticity items were also removed based on poor face validity as judged by the two habit experts. Specifically, these items were deemed ambiguous (e.g. ‘I have been sitting for a long time’) and difficult to understand (e.g. ‘not sitting makes me feel weird if I do not do it’) or were adjudged to capture perceived importance of sitting (‘I have no need to think about sitting’).

Conventional SRHI wording (e.g. ‘Sitting is something I do without having to consciously remember’) was simplified to aid comprehension (i.e. ‘I sit without having to consciously remember’). The six automaticity items entered into analysis are listed in Table 1.

**Table 1:** Principal component analysis of automaticity items

	Component 1: lack of awareness	Component 2: lack of control
Eigenvalue	2.81	1.32
Variance explained (%)	56.27	26.30
*Item loadings*		
“I sit without having to consciously remember”	.93	–
“I sit automatically”	.91	–
“I sit without thinking”	.88	–
“I would find it hard not to sit”	–	.89
“Sitting would require effort not to do”	–	.87

*N* = 194. Loadings < .40 not reported. The sixth automaticity item (“I sit before I realise I’m doing it”) was withheld from analysis as it loaded weakly on both components in an earlier iteration of the analysis

*Prompt adherence* was recorded using the Exertime software, which generated a date and time stamp each time an Exertime prompt was displayed and accepted by the participant. We did not directly assess whether movement breaks were taken, or for how long. Rather, we infer that a movement break was taken when an Exertime prompt was accepted.

### Data Management and Analyses

#### Principal Component Analysis of Automaticity Items

The structure of the six automaticity items, as measured at baseline and summed across both prompt conditions (*N* = 194), was assessed via principal component analysis (PCA), using maximum likelihood extraction and direct oblimin rotation. Analyses met sampling adequacy and sphericity assumptions. Component extraction was informed by parallel analysis [48], which provided, based on 100 random correlation matrices and 95th percentile eigenvalues, randomly generated benchmark eigenvalues for comparison with observed component eigenvalues. Item loadings were derived from the pattern matrix.

#### Changes in Automaticity

Following the PCA, scores for discrete automaticity components at each timepoint were generated using the means of constituent items. Intraclass correlations were used to evaluate the degree of change in automaticity scale scores. Differences in automaticity across time were tested using multilevel modelling, with level 1 specified as within-person change over the five timepoints and level 2 as between-person differences, in the *lme4* package of R [49, 50]. Missingness was imputed using the *mice* function following data pattern investigation [51]. A priori testing established that there was insufficient variability in automaticity strength between observed scale scores to include random effects for the third level (between scale scores), so the only random effects were for time within-person. Automaticity score was regressed onto the following: time; a dichotomous dummy variable indicating from which of the observed automaticity factors the score was derived; prompt adherence, expressed as the mean of daily prompts accepted; and the interaction between time and automaticity factor score (Tables 2 and 3).

**Table 2:** Descriptive statistics and bivariate correlations of automaticity scores and prompt adherence at all timepoints

		*Baseline* (*N* = 194)		*3 months* (*N* = 157)			*6 months* (*N* = 109)			*9 months* (*N* = 87)			*12 months* (*N* = 70)		
	Variable														
	*M* (SD)	LoA	LoC	LoA	LoC	PA	LoA	LoC	PA	LoA	LoC	PA	LoA	LoC	PA
*Baseline* (*N* = 194)															
Lack of awareness	5.66 (1.56)		.30*	.36*	.35*	− .11	.37*	.20*	− .11	.33*	.25*	.02	.21	.11	− .05
Lack of control	4.33 (1.73)			.16	.42*	.02	.17	.46*	− .03	.17	.46*	.05	.19	.40*	.04
*3 months* (*N* = 157)															
Lack of awareness	5.29 (1.62)				.45*	− .06	.59*	.39*	.06	.56*	.32*	.07	.59*	.38*	.07
Lack of control	4.12 (1.69)					− .12	.37*	.63*	− .07	.42*	.59*	.09	.48*	.67*	.14
Prompt adherence	5.46 (2.00)						− .06	.00	.76*	− .01	− .05	.69*	.10	.04	.61*
*6 months* (*N* = 109)															
Lack of awareness	5.26 (1.57)							.58*	− .09	.55*	.43*	− .08	.72*	.38*	− .05
Lack of control	3.99 (1.61)								− .01	.37*	.67*	.00	.51*	.70*	.02
Prompt adherence	4.88 (2.22)									.14	− .03	.89	.14	.12	.82*
*9 months* (*N* = 87)															
Lack of awareness	5.15 (1.54)										.44*	.18	.70*	.42*	.23
Lack of control	4.05 (1.63)											.00	.50*	.55*	.00
Prompt adherence	4.85 (2.32)												.12	.12	.92*
*12 months* (*N* = 70)															
Lack of awareness	4.99 (1.43)													.56*	.09
Lack of control	3.75 (1.64)														.10
Prompt adherence	4.43 (2.39)														−

*LoA* lack of awareness, *LoC* lack of control, *PA* prompt adherence

^*^p < .05

**Table 3:** Multilevel model regression estimates for testing change in automaticity over time

Dependent variable: automaticity	*b*	95% confidence interval
Intercept	5.80*	5.48 to 6.12
Time	− .16*	− .23 to − .10
Automaticity type: lack of control vs. lack of awareness as reference	− 1.24*	− 1.54 to − .95
Prompt adherence	− .02	− .05 to .02
Time × automaticity type	− .00	− .09 to .09

A total of 1940 observations (2 automaticity scale scores × 5 assessments from *N* = 194 with missingness = 706)

^*^p < .05

### Sample Size and Statistical Power

For PCA purposes, the ratio of participants (*N* = 194) to items (6) in the study was > 32:1, which exceeds the recommended minimum 10:1 ratio [52], indicating analytical adequacy. Power analysis for the multilevel models was estimated from 1000 Monte Carlo simulations using the *simr* package [53]. The study had a power of 86.9% (95% CI: 84.7 to 88.9%) for finding differences in change over time between automaticity factor scores for small effect sizes (*z* = .18).

## Results

### Principal Component Analysis of Automaticity Items

The six automaticity items were underpinned by two components (component 1: eigenvalue 3.38, 56.28% variance explained; component 2: eigenvalue 1.36, 22.62% variance). While five items each loaded strongly (≥ .85) on only one component, one item (‘I sit before I realize I’m doing it’) loaded relatively weakly on both (component 1 loading .48, component 2 loading .54). A second PCA, rerun excluding this item, also produced two components (component 1: eigenvalue 2.81, 56.27% variance explained; component 2: eigenvalue 1.32, 26.30% variance explained), with identical item loading patterns (see Table 1).

We deemed the first component, which captured three items (‘[I sit…] …without having to consciously remember’, ‘…automatically’, ‘…without thinking’), to capture a *lack of awareness of the initiation of sitting*, i.e. the extent to which a sitting episode can proceed outside of conscious attention. The second component, on which two items loaded (‘I would find it hard not to sit’, ‘Sitting would require effort not to do’), was judged to represent a *lack of control over not sitting*, that is, the perceived cognitive effort that would be incurred by attempting to inhibit sitting. At baseline, the two components correlated at *r* = .28, demonstrating their distinctiveness. Across the five measurement timepoints, interitem reliability for the lack of awareness scale ranged from *α* = .90 to .93 and from Spearman-Brown = .88 to .93 for the lack of control scale.

### Changes in Automaticity in Response to a Sitting-Reduction Intervention

Both automaticity subscales and prompt adherence declined over time. The automaticity subscales tended to be positively correlated across time. Prompt adherence scores tended to be strongly related across time but were not associated with automaticity variables.

The intraclass correlation for lack of awareness and lack of control was .45 (95% CI: .37 to .54) and .53 (95% CI: .46 to .61), and prompt adherence was .77 (95% CI: .72 to .81). These values indicate that the two observed automaticity subscales varied at both between- and within-person levels, with about half of the variability explained by between-person differences. Mean daily prompt adherence was mostly variable between participants, rather than changing over time, with more than two-thirds of variability at the between-person level.

**Fig. 1:**
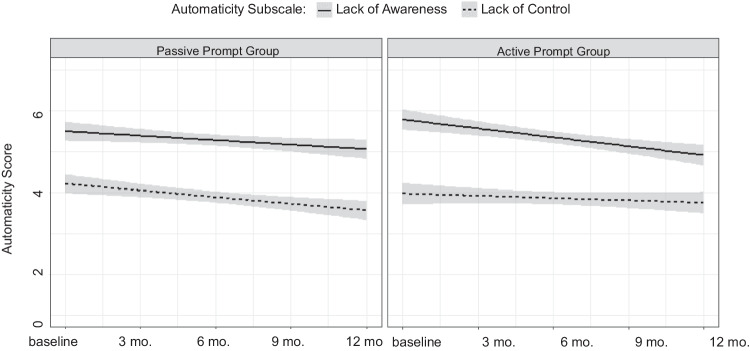
Changes in automaticity scores over time

Multilevel model results showed that overall, automaticity decreased over time. Additionally, there was a significant direct effect showing that, across timepoints, lack of awareness scores were stronger than lack of control automaticity scores. No moderation effect was found, demonstrating that changes over time did not differ between scales (Fig. 1).

## Discussion

Sitting is often portrayed as automatic, triggered by exposure to cues conducive to sitting (e.g. entering the office). Drawing on data from a 12-month intervention trial, we showed that the automaticity of sitting could be separated into two components, relating to the extent to which sitting episodes can proceed outside awareness, and a lack of control over or inhibiting sitting. At all timepoints, scores were higher for the lack of awareness automaticity subscale, suggesting that this is the more powerful of the two components in determining overall sitting habit strength. Scores on both components decreased over time in response to an intervention that aimed to break sitting habits by prompting office workers to take regular breaks. These results suggest that interventions should seek to disrupt sitting habits by increasing the conscious effort and attention required to sit and by providing greater opportunities to determine engagement whether to sit or adopt alternative postures.

Our analysis of six items designed to capture the automaticity of sitting extracted two components, representing a lack of awareness of the initiation of sitting (e.g. ‘I sit without automatically’) and a lack of control over whether to adopt non-seated alternatives (e.g. ‘I would find it hard not to sit’). This finding is noteworthy, because it empirically demonstrates that automaticity is multifaceted, rather than a unidimensional structure, and that the SRHI is potentially sensitive to discrete facets of automaticity. Although analyses typically extract single components from the SRHI [37, 38], our results echo studies that have uncovered multiple discrete components underlying self-reported automaticity [39]. Moreover, the number of factors, and their internal structure, observed in our data replicated findings from an analysis of SRHI responses relating to physical activity and those relating to snacking [39]. That study identified one factor tapping lack of awareness and another tapping lack of control, with identical loading patterns observed for the five items that we entered into our analysis. This suggests that the automaticity profile of sitting may resemble that of both physical activity and snacking behaviour.

Scores on both automaticity subscales declined over time in response to our two interventions, which involved prompting computer-based workers to take regular breaks from sitting, by temporarily blocking use of their workstation at regular intervals. All participants received one of two variants of a prompt-based intervention, and the lack of an inactive control group means that causality over decreases in the two automaticity indices cannot be definitively attributed to the intervention. More rigorous, controlled trials are needed to evaluate whether prompt-based interventions similar to those used in this study truly cause declines in sitting automaticity. Nonetheless, our findings are consistent with the possibility that prompting people to take regular breaks from computer-based sitting time may disrupt automatic sitting. Interestingly, there was no association between which of the two interventions participants received, and subsequent declines in automaticity, despite participants in one group only being able to accept or postpone the prompt, whereas others could also choose to dismiss it. If our findings are taken to indicate that prompt-based interventions can reduce sitting automaticity, then such effects may perhaps have arisen by enhancing the salience of sitting breaks, which in turn rendered taking a break—that is, *not* sitting—less cognitively effortful, and may have empowered people to experience greater control over their sitting time. More empirical work is needed to establish the most effective methods for reducing the two discrete components of automaticity we observed.

A key limitation of our study is that we did not record sitting time. While scores were higher on the lack of awareness automaticity subscale relative to the lack of control subscale, we cannot establish which of the two subscales has more influence on sitting time. Further work is needed to explore the impact of changes in automaticity components on sitting reduction more broadly. We also did not assess whether, and for how long, participants broke up their sitting when they accepted prompts. Yet, while it remains possible that participants may have simply switched to alternative seated activities (e.g. checking their phones) on activation of an Exertime prompt, primary analyses of the present dataset, published elsewhere, suggested that our computerized prompts initiated movement breaks as intended [46]. We did not obtain measures of motivation to reduce sitting, so we cannot estimate to what extent declines in sitting automaticity may have been attributable to participants’ purposeful attempts to reduce sitting, rather than the prompt compelling participants to take breaks potentially despite a lack of conscious motivation to do so. The absence of behaviour or motivation measures also means that we cannot establish how representative our sample is of office workers more broadly. Although there is no a priori reason to expect our sample to have had different baseline sitting patterns to any other desk-based workers, our participants volunteered to trial a novel sitting-reduction intervention. They may consequently have been more concerned about their sitting, and so more inclined to limit their sitting, than office workers more typically. The potential impact of our intervention on automaticity may therefore have been overestimated: office workers who lack motivation to reduce sitting time at work, because they view taking sitting breaks as obstructive to the pursuit of higher-priority work-related goals [54], may perhaps be less receptive to prompt-based interventions, and so might have experienced lesser decreases in sitting automaticity than we observed.

The factor analysis process also has limitations. The meaningfulness of extracted factors, as indicators of true underlying constructs, is dependent on the quality of the items entered into the analysis. We explored automaticity facets based on SRHI [33] items. Although developed to tap discrete dimensions of automaticity, the SRHI has been criticized for its lack of sensitivity to conceptually separable components [35]. It is possible that additional components may have emerged had we used an alternative measure, such as the Generalized Multifaceted Automaticity Scale [55], which proposes, a priori, a set of conceptually independent automaticity dimensions. Further work, using other measures, is needed to investigate the robustness of the two-dimensional solution that emerged from our analyses. Additionally, while factor analysis systematically identifies underlying statistical patterns, assigning labels to those patterns is an inherently subjective process. The factor that we deemed as indicative of a lack of awareness of initiating a sitting episode might alternatively be seen to capture cognitive efficiency, such that people are not required to think about initiating sitting. Similarly, we deemed the second factor to capture a lack of control over sitting, but this might alternatively be portrayed as a perception of greater effort being required to inhibit sitting. This speaks to the difficulty of reliably identifying closely inter-related, yet theoretically distinct components of automaticity. Indeed, some have argued that such distinctions are practically redundant, because participants fail to distinguish between different forms of automaticity when completing the SRHI [56]. Lastly, a sceptical reader might argue that the two-factor structure observed in this study, and previous research [39], simply reflects a distinction between SRHI items that reflect on sitting and those that focus on *not* sitting. Future work might examine whether the two-factor structure can be replicated when all SRHI items share the same directionality, by solely focusing on reflections on performing, or solely inhibiting performance of, a behaviour.

Sitting has been shown to be a non-reflective action, initiated automatically upon exposure to environments conducive to sitting [18, 41]. Automaticity is a multifaceted concept, and we showed that the automaticity of sitting can be separated into two components. These captured a lack of awareness, whereby people do not need to think before acting, and a lack of control, such that people find it difficult to inhibit sitting when encountering associated cues. Interventions seeking to disrupt automatic sitting behaviour should seek to increase conscious awareness of sitting, and create environments in which people are better able to exercise control over their sitting patterns.
